# Injury Rates in Iranian Taekwondo Athletes; a Prospective Study

**DOI:** 10.5812/asjsm.34877

**Published:** 2010-03

**Authors:** Vahid Ziaee, Seyed-Hessam Rahmani, Mohsen Rostami

**Affiliations:** 1 Sports Medicine Research Center, Tehran University of Medical Sciences, IR Iran

**Keywords:** Taekwondo, Martial arts, Sport injury, Concussion, Contusion, Athlete exposure

## Abstract

**Purpose:**

Taekwondo, as the most popular martial art among Iranian sportsmen, might lead to injury for the athletes of this sport during competitions. We decided to report the incidence rate of injuries sustained by the athletes of this sport during national competitions.

**Methods:**

All competitions of Iran national championship taekwondo league in 2006-2007 with 204 athletes were observed prospectively to detect the occurrence of injuries. The severity of injuries was classified into four groups (mild, moderate, severe, and critical) according to the involvement of medical care team in the contest, ability of the athletes to resume and duration of probable absence of injured athletes from future competitions and training sessions. Athlete-Exposure (A-E) was defined as the number of competitions multiplied by two. On this base, the rate of injury incidence per 1000 A-Es, the rate of injuries per time unit and the rate of injury occurred for each 100 athletes were considered as the major outcomes of this study.

**Results:**

Of totally 1,338 A-Es, 93 injuries were recorded during the competitions. The rate of injury incidence was found to be 69.5 injuries per 1000 A-Es and the rate of injuries per minute of competitions was 0.023 which corresponded to 23.3 injuries per 1000 minutes of competitions. 45.6 injuries were occurred for each 100 athletes during the course of competitions. The most frequent injuries were mild (68.8%) and critical injuries (24.7%), followed by moderate and severe injuries; 4.3% and 2.1%, respectively.

**Conclusion:**

The rate of injury we found was lower than that of western countries. In spite of finding the lower limbs as the most frequent place of injuries in other studies, we found the upper limbs as the most predisposed place of injuries which might be due to difference in the method of combat of Iranian athletes with other athletes.

## INTRODUCTION

Taekwondo, as a Korean martial art, is designated as an official sport field in the Olympic Games. This sportive field has the most athletes among different martial arts in the world. Although according to some studies the safeness of martial arts in comparison to non-contact sports have been studied ^[1, 2]^, similar to other martial arts the rate of injury incidence in this field seems to be higher than other non-contact sports ^[3]^. Not only competitions, but also training sessions could be assumed as potential conditions to cause injuries for athletes ^[4]^.

Epidemiologic studies have reported a wide range of injuries for participants in tournaments ^[4–6]^. It seems that findings in the matter of pattern and incidence of injuries in different studies vary according to the interests and popularity of martial arts among the people of the country where the study was performed and also the type of data authors used in their studies. Therefore, the picture might be different in Iran, a country with a relatively young population, where Taekwondo is supposed to be the most popular martial art.

High rate of Taekwondo-related injuries was the reason of establishing some rules which according to them using safety equipments like head and chest guards during matches has become mandatory. Nevertheless, injuries are still one of the common problems of taekwondo competitors. Injuries might lead to disruption of competitions or even referral of injured athletes to hospitals immediately during competitions ^[7]^. The incidence rate of injury for men attending in different taekwondo competitions was reported in a range of 20.5 to 168.4 injuries for each 1000 men ^[4, 8–11]^. According to the existed evidence, the lower limbs is the most prevalent place of injury followed by the head, neck and vertebral column ^[7, 8,10–14]^. Contusion and sprain were also have been found as the most prevalent types of injuries sustained ^[4, 7–18]^.

Considering the role of physical abilities and fitness of players in occurrence of injuries, finding a different pattern of injuries among the taekwondo competitors of countries with higher level of taekwondo champions in relation to players of lower level champions could be anticipated.

Concerning relatively high percentages of injuries reported in this field in former studies and existence of abundant advocates and athletes involved in this field of sport in Iran, the present study was carried out to find the pattern and rate of taekwondo-related injuries in Iranian athletes.

## METHODS AND SUBJECTS

This prospective and descriptive study was performed through data gathering from the national championship taekwondo league of males carried out from July 2006 to March 2007 in Iran. 204 male black-belt taekwondo competitors attended in this tournament. According to the weight of players, they were divided into eight groups as the athletes in the same groups could compete with each other. Regarding the standard rules of taekwondo, the competitions were designed in three periods (each one lasted for 2 minutes) that were broken by two rest times (each lasted for 30 seconds). The protective gears were allowed to be used by the players according to standard international protocols.

During the contests, injuries which occurred were totally recorded using a questionnaire. The questionnaire was extracted from the standard questionnaire of sports injury registration which its reliability had been confirmed previously. The data asked for in the questionnaire could be classified in 2 types; some questions were focusing on the demographic information of athletes including, name, age, weight, duration of taekwondo playing and also color of the belt and other questions were focusing on severity and type of injuries which the players sustained during competitions (including the existence of concussion, sprain, contusion, cramp, nose bleeding, dislocation, etc.). The first part of questionnaire was filled out by an interviewer and the second part was completed a specialist physician, according to observational findings of injures and their follow-up.

In this study, injury was defined as any affliction led to medical team involvement during or after the competition. The injuries were categorized in following 4 groups according to their severity; mild injuries were defined as the injuries which led to suspension of the contest for participation of medical care team in accordance with the request expressed by the competitor or the referee of the match; moderate injuries were defined as the injuries which led to stoppage of the game without possibility of resuming the competition; severe injuries were defined as the injuries which not only led to stoppage of the game, but also resulted in the absence of the competitor in the future training or matches up to one week; and critical injuries were defined as the injuries which not only led to stoppage of the competition, but also resulted in the absence of the competitor in the future training or matches for more than 1 week. In the matter of concussion and head injuries, Colorado Classification criteria were used to classify the severity of injuries ^[19]^.

Therefore, due to the method of classification used in this study, concussion which resulted in prohibition of the attendance of athletes in official competitions up to 1 month after sustaining injury was placed in the critical injuries category. Dislocation of fingers which were fixed during the match while the athletes could continue the competition was placed in mild injuries category. However, simultaneous dislocation and fracture of bones which occurred due to one circumstance were marked as two critical injuries. The injured athletes were also followed if they were referred to hospital for more specific evaluations; in addition, attendance of injured athletes in contests was asked through calling to athletes or making an interview. Not only all of the injured athletes confirmed the accomplishment of the study through signing the informed consent provided by the researchers but also, this study was approved by the Ethical Committee of Tehran University of Medical Sciences.

Due to the participation of two athletes in each competition, the Athlete-Exposure (A-E) was defined as the number of the matches multiplied by two. In addition, the incidence rate of injury per each 1000 A-E was calculated through multiplication of 1000 by the number of injuries dividing to the number of A-E^[8]^. Rate of injury per time unit was also measured through multiplication of 1000 by the number of injuries dividing to total time of exposures. Furthermore, the rate of injury occurred for each "100 athletes" was also calculated. The data obtained were analyzed using SPSS Software for Windows 16.0 (SPSS Inc., IL, US).

## RESULTS

The mean age of athletes in Iran championship league was 22.9 years (range, 17-32 years). Totally, and during the course of the league, 669 contests were established, therefore the A-E in the tournament was found to be 1,338. The competitions of the league lasted for 3,992 minutes. Sixty-four athletes (31.4%) sustained injuries during the course of competitions; in this regard 93 injuries were recorded. Mean age of injured athletes was 22.7 years (range, 17-30 years) and mean weight of injured athletes was 68.3kg (range: 52-90). The rate of injury incidence was found to be 69.5 injuries per 1000 A-Es and the rate of injuries per minute of matches was 0.023 which equals to 23.3 injuries per 1000 minutes of competitions. As data shows, 45.6 injuries occurred for each 100 athletes. Of totally 93 recorded injuries, 40 injuries (43.0%) were occurred in the upper limbs (32.1 injuries per 1000 A-Es) and 39 injuries (41.9%) were placed in the lower limbs (29.1 injuries per 1000 A-Es). The rate of injuries occurred in the head and neck and trunk of the athletes were equal; 7 injuries (7.5%) were placed in each of the mentioned parts of the body (5.2 injuries per 1000 A-Es). The rate of injury incidence in each part of the body is shown in Table 1. Three most prevalent injuries were contusion, sprain, and cutaneous ruptures which occurred in 30.6, 7.5 and 6.7 of players per 1000 athletes, respectively. Three concussions were registered which all three were categorized in first degree of Colorado Classification criteria (no loss of memory and consciousness). Of nine fractures observed, eight were placed in the upper limbs and only one of them was occurred in the lower limb. Regarding the incidence of dislocation, findings show that all 7 cases of dislocations reported were occurred in the upper limb. In the matter of concurrent injuries, it was found that three cases of fractures and dislocations were occurred simultaneously due to the same circumstances.

**Table 1:** Distribution of injury incidence in each region and site of body per 1000 Athlete-Exposures

Anatomical region of body	Site of injury	Number of injuries	Rate of injuries per 1000 athlete-exposure
**Head and Neck**	Head	3	2.24
	Lip	3	2.24
	Nose	1	.74
	Spinal column	2	1.49
**Trunk**	Shoulder	1	.74
	hip	4	2.98
	Elbow	3	2.24
	Forearm	10	7.47
**Upper limbs**	Wrist	1	.74
	Palm	10	7.47
	Hand fingers	16	11.95
	Thigh	17	12.7
	Knee	5	3.73
	Leg	7	5.23
**Lower Limbs**	Ankle	5	3.73
	Sole	3	2.24
	Foot fingers	2	1.49
**Total**		93	60.5

Sixty-four injuries (68.8%) were categorized in the group of mild injuries, 4 injuries (4.3%) were recorded as moderate injuries, two injuries (2.1%) were found as severe injuries and finally 23 injuries were reported as critical injuries (24.7%). Most injuries occurred in the third round of the matches including 46 injuries (49.5%). Forty (43.1%) and 7 (7.5%) injuries recorded in the second and first round of the matches, respectively.

## DISCUSSION

As the findings show, the rate of injuries (69.5 per 1000 A-Es) in Iranian athletes is lower than the rates reported from most of European and American countries ^[4, 8,9,10, 14]^. Although Beis et al ^[11]^ found 20.5 injuries per 1000 A-Es (lowest reported rate of injury in the literature) and also Pieter et al. reported 51.3 injuries per 1000 A-Es for taekwondo athletes, it seems the rate was found in Iran could be considered lower than the mean of injury rates reported previously ^[3]^. We found that the rate of injury per 1000 minutes was 23.3 injuries. This figure has been less reported in previous studies and we can not compare this finding with similar measurements in other studies; while including this measurement in analyses of sport injuries has been recommended previously ^[20]^. The skill, physical preparation and experience of athletes might be assumed as impressive factors in the probability of occurrence, severity, and the prognosis of injuries which the competitors incur ^[8, 10,21]^. In view of the high level and elite players attend in Iranian championship league the lower rates of injuries could be justified.

The pattern of injuries in Iranian taekwondo championship league is also somehow different in comparison to previous studies. In former studies, lower limbs were determined as the most prevalent anatomic region of the body to sustain injury during competitions followed by the head and neck and the upper limbs^[9–14, 22]^; however we found upper extremities injuries as the most prevalent anatomical place of injuries. This difference might be due to the more involvement of the upper limbs in the type of contest Iranian athletes participate. The second most common anatomic region of injuries in our study was the lower extremities, followed by head and neck and trunk injuries. Similar to other studies ^[3, 9,11, 13]^, contusion was found to be the most frequent injury in taekwondo competitions which could be related to the property of contact sports.

Low rate of concussion occurrence in competitions might imply to the effectiveness of protective equipments in reduction of such injuries. Although the role of protective gears in improvement of players’ health has also been mentioned in other studies ^[5]^ there is evidence which imply that researchers are not still sure regarding the beneficial effects of protection devices and safety equipments in taekwondo competitions ^[14, 23]^. Nevertheless, the rate of concussion found in this study was lower than the measures reported previously. This finding and high rate of the upper limbs injuries might be due to the skill of Iranian taekwondo athletes in protection of their head and neck using their upper limbs which led to eight fractures in the upper limbs of the total of nine fractures detected.

Most frequent time of injuries was the third round of competitions followed by the second and first time periods. This finding could be in accordance with the tiredness and exhausted energy of players during the last minutes of competitions. This is the first time, which the time period of injuries has been evaluated and there is no previous report in this regard; thus more evaluation and studies are required to determine the presence of any relation between the time periods of a competition and the likelihood of occurring injuries.

As it was mentioned by Koh et al ^[7]^ we also found that most of injuries were categorized as mild injuries; although the way of scaling or categorization was used by aforementioned authors was different from ours. The rate of moderate (26.5%) and sever (25.5%) injuries reported by Koh et al ^[7]^ were higher than the rates we found (4.3% and 2.1%, respectively). The high rate of critical injuries and low rate of severe and moderate injuries we found in the present study might show that taekwondo players are less interested in discontinuing the competitions and more often the matches were discontinued due to critical injuries. This finding should be much arousing for medical care teams of competitions since they can prevent the athletes from continuing the contest and in this way save the healthiness of players in long term.

A limitation of our study was that we only included male athletes. Also, the small sample size which was investigated in this study might be noted as another limitation. In addition, according to some evidence, education level of players and referees concerning prevention from injuries during the competitions might decrease the rate of injuries ^[12, 13,24]^; however the level of education of the participators in the competitions was not assessed.

It seems that to clarify the role of variable factors which may increase the chance of occurring injuries, performing prospective controlled trials to compare the effectiveness of different factors in the incidence rate of injury during taekwondo competitions is required.

## CONCLUSION

Our findings show that the pattern and incidence of injuries during the taekwondo competitions in Iran could not be compared with reported data from other countries in a favorable way. Although some speculations which imply to differences in skill and method of combating of Iranian taekwondo players might be presented in aim of justifying this discrepancy, however more controlled investigations are needed for more precise recommendations in this regard.

More focusing on the time of injury happening not only to find any probable relation between the round of a match and the incidence of injuries, but also tightening the protective rules for athletes in the last round of contests if the mentioned relation was proved.
